# Advanced chronic kidney disease increases the odds of ERCP adverse events but not post-ERCP pancreatitis: a propensity-matched analysis of the US Collaborative Network

**DOI:** 10.1007/s00464-025-12323-x

**Published:** 2025-10-22

**Authors:** Hussein Baydoun, Azizullah Beran, Daryl Ramai, Vikram R. Rajagopalan, Aladdin Said Dahbour, Mina Batarseh, Ujwala Pamidimukkala, Eugene Nwankwo, Islam Mohamed, Clive Jude Miranda, Hisham Wehbe, Oluwafisayo Adebiyi, Indira Bhavsar-Burke, John J. Guardiola, Itegbemie Obaitan

**Affiliations:** 1 Department of Medicine, Indiana University School of Medicine, Indianapolis, IN USA; 2 Division of Gastroenterology and Hepatology, Indiana University School of Medicine, Indianapolis, IN 46202 USA; 3 Division of Gastroenterology and Hepatology, Brigham and Women’s Hospital, Harvard Medical School, Boston, MA USA; 4 Division of Gastroenterology and Hepatology, Mayo Clinic Rochester, Rochester, MN USA; 5 Department of Medicine, Fairview Cleveland Clinic Hospital, Cleveland, OH USA; 6 Division of Gastroenterology, Creighton University Medical Center-Bergan Mercy, Omaha, NE USA; 7 Division of Gastroenterology, Digestive Disease Institute, Cleveland Clinic, Cleveland, OH USA; 8 Division of Nephrology, Indiana University School of Medicine, Indianapolis, IN USA; 9 Division of Gastroenterology and Hepatology, University of Texas Southwestern Medical Center, Dallas, TX USA

**Keywords:** Chronic kidney disease, End-stage renal disease, Endoscopic retrograde cholangiopancreatography, Post-ERCP pancreatitis, Post-procedure bleeding; cholangitis

## Abstract

**Background:**

Patients with chronic kidney disease (CKD) are at an increased risk choledocholithiasis, requiring intervention with endoscopic retrograde cholangiopancreatography (ERCP). Data on ERCP-related adverse events in this population is limited, hence this study.

**Methods:**

This retrospective cohort study utilized the TriNetX database to assess the odds of ERCP-related adverse events in patients with stages 4 and 5 chronic kidney disease, as well as end-stage renal disease (ESRD) on dialysis. Primary outcomes were ERCP-related pancreatitis (PEP), bleeding, cholangitis, and perforation. Secondary outcomes were failure to extubate/new post-procedure intubation, intensive care unit (ICU) admissions, and all-cause mortality.

**Results:**

After propensity score matching, our study included 4450 patients in the aCKD cohort and 4450 patients in the matched control cohort who underwent ERCP. Patients with aCKD had an increased odds of bleeding (OR 2.1, p < 0.001), cholangitis (OR 1.6, p < 0.001), ICU admissions (OR 2.2, p < 0.001), intubation (OR 3.0, p < 0.001) and mortality (OR 1.8, p < 0.001) compared to those with normal renal function. The odds of PEP (OR 1.1, p = 0.542) and perforation (OR 1.3, p = 0.528) were statistically similar between the two cohorts. No subgroup differences in ERCP-related AE were found except for increased ICU admissions odds in ESRD patients.

**Conclusion:**

aCKD patients are a demonstrably high-risk group for certain ERCP-related AEs but not for PEP or perforation, a finding that may change the previous widespread perception of increased PEP risk in this population. Additional studies are needed to validate our findings and investigate potential interventions to improve clinical outcomes in this high-risk population.

**Supplementary Information:**

The online version contains supplementary material available at 10.1007/s00464-025-12323-x.

Chronic kidney disease affects an estimated one in seven adults in the United States, approximately 36 million Americans [1]. Of these, 0.4% of patients have stage 4 chronic kidney disease, 0.2% have stage 4 chronic kidney disease and nearly 808,000 people in the United States (US) have end-stage renal disease (ESRD), with 69% of ESRD patients estimated to be on dialysis [2].

Patients with ESRD are at increased risk of cholestasis and gallstone formation, which may in turn lead to choledocholithiasis and or cholangitis [3, 4]. Endoscopic retrograde cholangiopancreatography (ERCP) is an advanced endoscopic procedure used for the definitive management of biliary and pancreatic disorders and is associated with adverse events (AEs) that can be potentially life-threatening such as post-ERCP pancreatitis (PEP) and post-sphincterotomy bleeding [5]. Few studies [6, 7] have explored ERCP-related adverse events (AEs) in patients with ESRD patients only, but there is a gap in the literature regarding the risks of ERCP in patients with advanced chronic kidney disease (aCKD) which encompasses stages 4 and 5 chronic kidney disease as well. Therefore, to address this gap, we conducted a propensity-matched cohort study in the US to better characterize ERCP-related complications in this population. Our a priori hypothesis was that patients with aCKD have higher odds of having ERCP-related AEs compared to the general population.

## Methods

### Study design and data source

This large, population-based, retrospective cohort study was conducted using the U.S. Collaborative Network in the TriNetX platform (Cambridge, MA, USA). TriNetX is a federated multicenter research network that provides real-time access to a deidentified dataset from participating healthcare organizations' electronic health records (EHR). Clinical data is obtained directly from EHRs and supplemented by a built-in natural language processing system, which extracts relevant variables from clinical documents. Rigorous quality control is applied at the point of data extraction to ensure accuracy before inclusion in the database. The platform displays only aggregate counts and statistical summaries to protect patient privacy, keeping data de-identified at all stages, with additional anonymity safeguards such as masking patient counts below 11. Further details of the TriNetX network are described in previous studies [8–10]. We followed the Strengthening the Reporting of Observational Studies in Epidemiology (STROBE) reporting guideline [11].

### Study participants, inclusion and exclusion criteria

A real-time search and analysis of the U.S. Collaborative Network on the TriNetX platform was conducted from October 2015 when the use of ICD-10 codes was mandated by the Centers for Medicare and Medicaid (CMS), through to March 24, 2025. Our study had two cohorts. The advanced chronic kidney disease (aCKD) cohort included all adults (≥ 18 years) with a diagnosis of chronic kidney disease (CKD) stage 4 defined as an eGFR of 15–29 ml/min/1.73 m^2^, CKD stage 5 defined as an eGFR of 15 ml/min/1.73 m^2^ or less for three months for now and end-stage renal disease (ESRD) patients on dialysis who underwent ERCP. These patient groups were identified by International Classification of Diseases-10 (ICD-10) codes and the Current Procedural Terminology (CPT) codes. Details on data sources and diagnosis codes used for patient selection and outcomes (according to predefined ICD-10, CPT, and RxNorm codes) are described further in Supplementary Table 1. To ensure ERCP occurred after the aCKD diagnosis, TriNetX's functionality for defining index events and excluding prior outcomes was applied, including only patients who received ERCP following the aCKD ICD-10 code. The control cohort consisted of all adults (≥ 18 years) who underwent ERCP in the same period and did not have an ICD-10 code for any CKD diagnosis. In both cohorts, we excluded patients less than the age of 18, patients who had ERCP prior to the diagnosis of aCKD and patients who had a documented diagnosis of stages 1, 2, or 3 chronic kidney disease.

### Study outcomes

The primary outcomes of interest were the 7-day post-procedure odds of ERCP-related adverse events such as post-ERCP pancreatitis (PEP), post-procedural bleeding and intraprocedural perforation, and post-procedural cholangitis. Secondary outcomes of interest were the 7-day post-procedure odds of intensive care unit (ICU) admissions, failure to extubate and/or new post-procedure intubation for cardiorespiratory or mental status reasons 48 h post-procedure, and 30-day all-cause mortality. All ICD-10 codes used to identify these outcomes are listed in Supplementary Table 1.

### Statistical analysis

All statistical analyses were performed using the real-time analytics feature, TriNetX Live (TriNetX LLC, Cambridge, MA), on the TriNetX platform. Baseline characteristics of study cohorts were reported using means, standard deviations, and proportions. Propensity score matching was done to balance confounding variables between groups thereby mitigating selection bias, increase precision and to avoid overfitting issues that sometimes arise with the use of traditional regression models. One-to-one (1:1) propensity score matching was performed for demographics (age, sex and ethnicity), comorbidities (diabetes mellitus, hypertension, ischemic heart disease, cirrhosis, thrombocytopenia and hereditary factor VIII deficiency) and relevant medications (aspirin, clopidogrel, ticagrelor, prasugrel, warfarin, apixaban, rivaroxaban, and dabigatran). The TriNetX platform generates propensity scores for individual subjects through logistic regression analysis based on the selected covariates. Patients were matched using a greedy nearest-neighbor algorithm with a caliper width of 0.1 pooled standard deviations, which was determined optimal. To prevent bias from the nearest-neighbor algorithm, TriNetX randomizes row order. After PSM, outcome measures were calculated and expressed as odds ratios (OR) with 95% confidence intervals (CIs). Statistical significance was defined as a two-sided p-value < 0.05.

## Results

### Baseline characteristics

We identified 4532 patients diagnosed with aCKD and 167,823 control patients who underwent ERCP during the study period. The aCKD cohort had a higher proportion of older population (64.0 vs. 58.6, p < 0.001), male patients (53.1% vs. 41.7%, p < 0.001), African-American or Black patients (15.8% vs. 8.7%, p < 0.001), type 2 diabetes mellitus (45.5% vs. 17.2%, p < 0.001), hypertension (74.7% vs. 40.6%, p < 0.001), ischemic heart disease (33.6% vs. 11.7%, p < 0.001), cirrhosis (19.4% vs. 4.0%, p < 0.001), thrombocytopenia (24.5% vs. 5.9%, p < 0.001), and hereditary factor VIII deficiency (0.2% vs. 0.0%, p < 0.001) compared to the control cohort. Additionally, a greater proportion of patients in the aCKD cohort were on antiplatelets: aspirin (44.7% vs. 22.2%, p < 0.001), clopidogrel (11.4% vs. 3.8%, p < 0.001), ticagrelor (1.2% vs. 0.4%, p < 0.001) and prasugrel (0.4% vs. 0.2%, p < 0.001), and on anticoagulants: warfarin (11.5% vs. 3.9%, p < 0.001) and apixaban (8.6% vs. 3.7%, p < 0.001, Table 1).

**Table 1:** Baseline patient characteristics before and after propensity-score matching (PSM)

	Before PSM			After PSM		
	aCKD	Control	p-value	aCKD	Control	p-value
Total number of patients, n	4455	161,463		4450	4450	
Demographics						
Age (mean ± SD)	64.0 ± 15.0	58.6 ± 18.5	** < 0.001**	64.0 ± 15.0	65.2 ± 14.7	** < 0.001**
Male (%)	53.1	41.7	** < 0.001**	53.1	54.5	0.180
Race						
White (%)	63.2	70.1	** < 0.001**	63.3	65.1	0.073
African American or Black (%)	15.8	8.7	** < 0.001**	15.7	15.6	0.884
Asian (%)	4.6	3.9	0.079	4.6	4.7	0.802
Ethnicity						
Hispanic or Latino (%)	10.8	9.9	**0.035**	10.9	9.8	0.109
Comorbidities						
Diabetes mellitus (%)	45.5	17.2	** < 0.001**	45.4	46.4	0.317
Hypertension (%)	74.7	40.6	** < 0.001**	74.7	75.3	0.509
Ischemic heart disease (%)	33.6	11.7	** < 0.001**	33.5	32.9	0.559
Cirrhosis (%)	19.4	4.0	** < 0.001**	19.4	19.4	0.979
Thrombocytopenia (%)	24.5	5.9	** < 0.001**	24.4	23.9	0.569
Hereditary factor VIII deficiency (%)	0.2	0.0	** < 0.001**	0.2	0.2	1
Medications						
Aspirin (%)	44.7	22.2	** < 0.001**	44.6	44.3	0.293
Clopidogrel (%)	11.4	3.8	** < 0.001**	11.3	10.6	0.733
Ticagrelor (%)	1.2	0.4	** < 0.001**	1.2	1.1	0.266
Prasugrel (%)	0.4	0.2	**0.001**	0.4	0.2	0.177
Warfarin (%)	11.5	3.9	** < 0.001**	11.5	10.6	0.187
Apixaban (%)	8.6	3.7	** < 0.001**	8.6	7.6	0.095
Rivaroxaban (%)	2.3	2.1	0.332	2.3	2.1	0.386
Dabigatran (%)	0.4	0.3	0.395	0.4	0.2	0.177

The values in bold are *p*-values that are less than 0.05, suggesting statistically significant differences between both groups

*aCKD* advanced chronic kidney disease (CKD4–5 and ESRD), *PSM* propensity-score matching, *SD* standard deviation

After propensity score matching was carried out, we had a total of 8900 patients who underwent ERCP (4450 patients in the aCKD cohort and 4450 patients in the matched control cohort). Propensity score matching was successful for all measures except age (aCKD patients were younger 64.0 ± 15.0 vs. 65.2 ± 14.7, p < 0.001). A complete list of all demographic parameters, comorbidities, and relevant medications can be found in Table 1.

### Primary outcomes

**Fig. 1:**
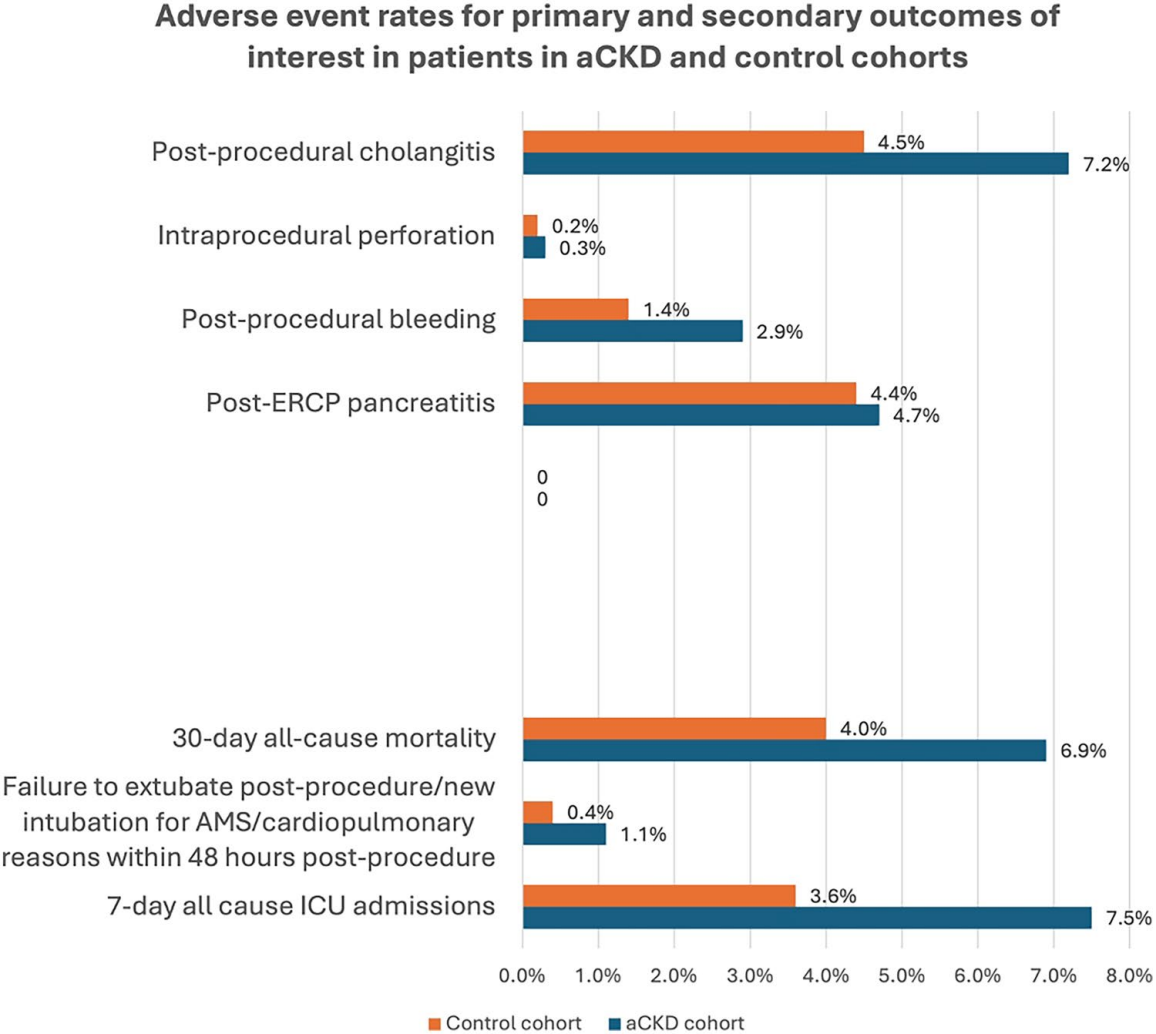
Adverse event rates for primary and secondary outcomes of interest in patients in aCKD and control cohorts

After PSM, a total of 101 patients (2.9%) in the aCKD cohort had bleeding after ERCP compared to 55 patients (1.4%) in the control group. The odds of post-procedural bleeding was higher in the aCKD cohort compared to the control cohort (OR 2.1, 95% CI 1.5–2.9, p < 0.001, Table 2). A total of 262 patients (7.2%) had cholangitis after ERCP compared to 170 patients (4.5%) in the control group. The odds of post-procedural cholangitis was higher in the aCKD cohort compared to the control cohort (OR 1.6, 95% CI 1.3–2.0, p < 0.001). However, there were no statistically significant differences in PEP (4.7% vs. 4.4%; OR 1.1, 95% CI 0.9–1.3, p = 0.542) and intraprocedural perforation (0.3% vs. 0.2%; OR 1.3, 95% CI 0.6–3.0, p = 0.528) between the two cohorts (Fig. 1; Table 2).

**Table 2:** ERCP-related adverse events: primary and secondary outcomes of interest

Outcomes of interest	OR, 95% CI	p-value
Primary outcomes of interest		
Post-ERCP pancreatitis	1.1 (0.9–1.3)	0.542
Post-procedural bleeding	2.1 (1.5–2.9)	** < 0.001**
Intraprocedural perforation	1.3 (0.6–3.0)	0.528
Post-procedural cholangitis	1.6 (1.3–2.0)	** < 0.001**
Secondary outcomes of interest		
7-Day intensive care unit admissions (all-cause)	2.2 (1.8–2.7)	** < 0.001**
Failure to extubate post-procedure/new intubation for AMS/cardiopulmonary reasons within 48 h of the procedure	3.0 (1.7–5.3)	** < 0.001**
30-Day mortality (all-cause)	1.8 (1.5–2.2)	** < 0.001**

The values in bold are *p*-values that are less than 0.05, suggesting statistically significant differences between both groups

*ERCP* endoscopic retrograde cholangiopancreatography, *aCKD* advanced chronic kidney disease (CKD4–5 and ESRD), *AE* adverse events, *CI* confidence interval, *OR* odds ratio, *AMS* altered mental status

### Secondary outcomes

The aCKD cohort had significantly higher odds of 7-day all-cause ICU admissions (OR 2.2, 95% CI 1.8–2.7, p < 0.001), intubations within 48 h of the procedure (OR 3.0, 95% CI 1.7–5.3, p < 0.001) and 30 days all-cause mortality (OR 1.8, 95% CI 1.5–2.2, p < 0.001) following ERCP compared to the matched cohort control (Table 2).

### Subgroup analysis

#### Baseline characteristics

We identified 2490 patients diagnosed with ESRD on dialysis and 1003 patients who underwent ERCP during the study period. The ESRD cohort had a higher proportion of male patients (56.9% vs. 41.9%, p < 0.001), African-American or Black patients (16.4% vs. 10.6%, p < 0.001), type 2 diabetes mellitus (45.6% vs. 41.3%, p = 0.023), cirrhosis (27.0% vs. 12.0%, p < 0.001), thrombocytopenia (26.6% vs. 12.0%, p < 0.001), and hereditary factor VIII deficiency (0.2% vs. 0.0%, p = 0.045) compared to the CKD4–5 cohort. Additionally, a greater proportion of patients in the CKD4–5 cohort were on prasugrel (0.4% vs. 1%, p = 0.034), and on dabigatran (0.4% vs. 1%, p < 0.034, Table 3).

**Table 3:** Baseline patient characteristics before and after propensity-score matching (PSM), subgroup analysis

	Before PSM			After PSM		
	ESRD + dialysis	CKD4–5	p-value	ESRD + dialysis	CKD4–5	p-value
Total number of patients, n	2490	1003		783	783	
Demographics						
Age (mean ± SD)	60.0 ± 15.0	73.4 ± 13.0	** < 0.001**	70.6 ± 12.1	70.4 ± 12.6	0.781
Male (%)	56.9	46.9	** < 0.001**	51.5	51.1	0.879
Race						
White (%)	61.7	69.2	** < 0.001**	67.6	66.0	0.520
African American or Black (%)	16.4	10.6	** < 0.001**	12.4	12.4	1
Asian (%)	4.9	6.0	0.207	5.5	5.6	0.912
Ethnicity						
Hispanic or Latino (%)	12.8	7.0	** < 0.001**	9.2	8.0	0.418
Comorbidities						
Diabetes mellitus (%)	45.6	41.3	**0.023**	45.8	44.1	0.477
Hypertension (%)	71.4	72.8	0.422	74.2	73.1	0.606
Ischemic heart disease (%)	34.2	36.9	0.137	38.4	36.8	0.498
Cirrhosis (%)	27.0	12.0	** < 0.001**	13.8	13.5	0.883
Thrombocytopenia (%)	26.6	12.0	** < 0.001**	16.9	14.7	0.239
Hereditary factor VIII deficiency (%)	0.4	0	**0.045**	0	0	1
Medications						
Aspirin (%)	39.1	35.5	0.052	35.1	36.5	0.562
Clopidogrel (%)	9.7	11.0	0.261	11.1	10.9	0.872
Ticagrelor (%)	1.2	1.1	0.957	1.4	1.3	0.826
Prasugrel (%)	0.4	1.0	**0.034**	1.3	1.3	1
Warfarin (%)	8.6	9.8	0.301	11.0	9.8	0.456
Apixaban (%)	8.8	9.0	0.827	8.7	8.8	0.929
Rivaroxaban (%)	2.3	3.1	0.191	2.7	2.7	1
Dabigatran (%)	0.4	1.0	**0.034**	1.3	1.3	1

The values in bold are *p*-values that are less than 0.05, suggesting statistically significant differences between both groups

*CKD* chronic kidney disease, *PSM* propensity-score matching, *SD* standard deviation

After propensity score matching was carried out, we had a total of 1566 patients who underwent ERCP (783 patients in each cohort). Propensity score matching was successful for all measures. A complete list of all demographic parameters, comorbidities, and relevant medications can be found in Table 3.

#### Outcomes

**Fig. 2:**
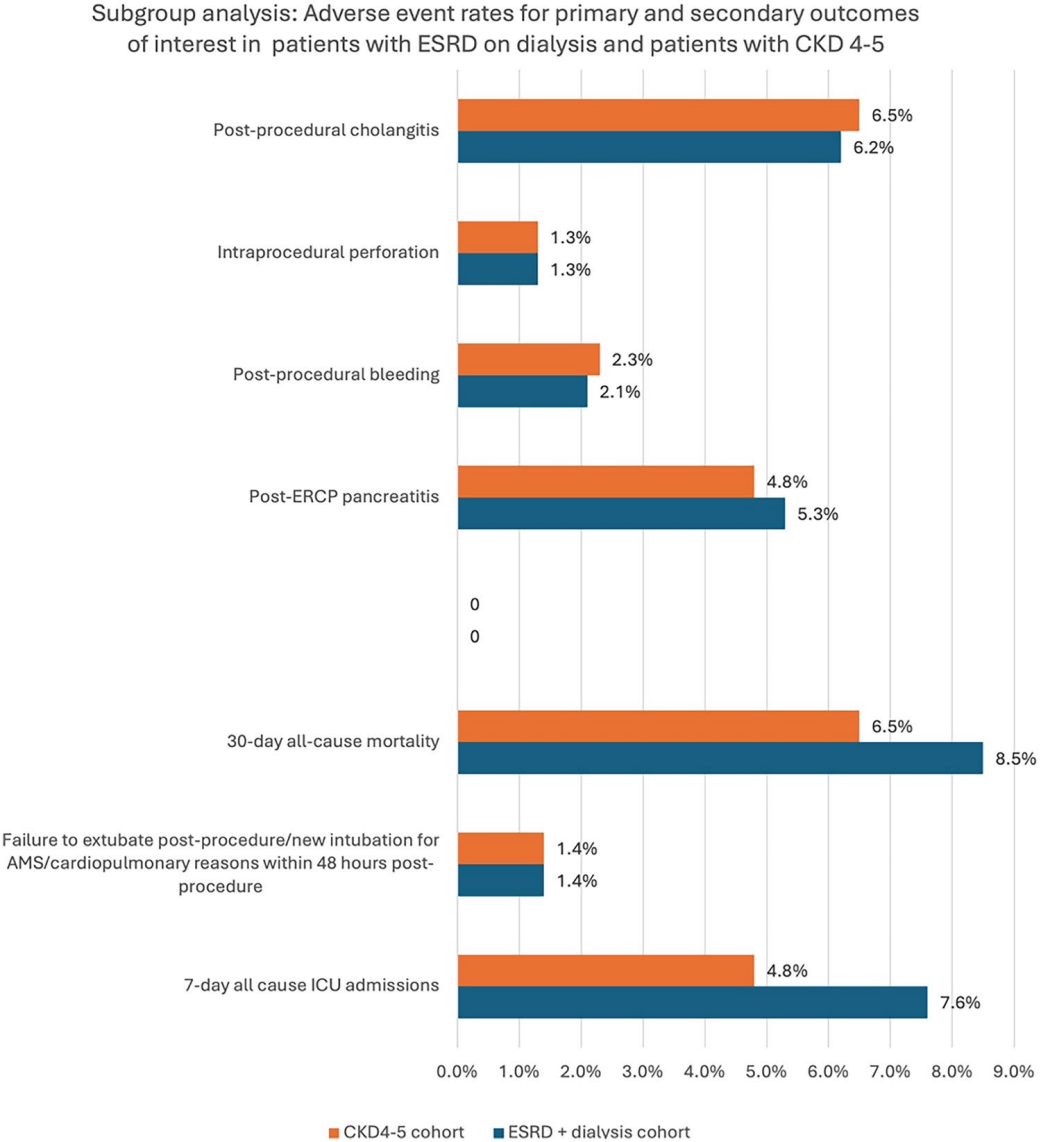
Adverse event rates for primary and secondary outcomes of interest in patients in ESRD on dialysis and CKD4–5 cohorts, subgroup analysis

After PCM, the odds of 7-day ICU admission post-ERCP were higher in the ESRD cohort compared to the CKD4–5 group (OR 1.6, 95% Cl 1.0–2.6, p = 0.043). However, there were no statistically significant differences in all other outcomes between the two cohorts (Fig. 2; Table 4).

**Table 4:** ERCP-related adverse events: primary and secondary outcomes of interest in our subgroup analysis

Outcomes	OR, 95% CI	p-value
Primary outcomes of interest		
Post-ERCP pancreatitis	1.1 (0.6–1.8)	0.716
Post-procedural bleeding	0.9 (0.5–1.9)	0.845
Intraprocedural perforation	1.0 (0.4–2.4)	1.00
Post-procedural cholangitis	1.0 (0.6–1.5)	0.90
Secondary outcomes of interest		
7-Day intensive care unit admissions (all-cause)	1.6 (1.0–2.6)	**0.043**
Failure to extubate post-procedure/new intubation for AMS/cardiopulmonary reasons within 48 h of the procedure	1.0 (0.4–2.4)	1.00
30-Day mortality (all-cause)	1.3 (0.9–2.0)	0.144

The value in bold is *p*-value that is less than 0.05, suggesting statistically significant differences between both groups

*ERCP* endoscopic retrograde cholangiopancreatography), *AE* adverse events, *CI* confidence interval, *OR* odds ratio, *AMS* altered mental status

## Discussion

This study was conducted with reported procedural outcomes from the TriNetX database, a collection of large multicenter, nationally representative real-world data. This is the largest study to our knowledge investigating ERCP-related adverse events (AEs) in patients with CKD4, CKD5 and ESRD patients on hemodialysis—collectively referred to as advanced chronic kidney disease (aCKD) in this study, with data that has undergone propensity score matching to mitigate the selection bias that is prevalent in retrospective studies.

PEP is the most common post-ERCP related adverse event with a reported incidence of 10.2% [12]. Interestingly, our data doesn’t show an increased odds of PEP in our cohort compared to the control population, which is in contrast with previously reported data. The 2019 published European Society for Gastrointestinal Endoscopy guidelines [13] suggest there may be an increased risk of PEP in patients with ESRD, a statement based on two studies. The larger study by Sawas et al. [7] was a retrospective study looking at 2011 to 2013 data from the nationwide inpatient sample, with the vast majority of patients being controls. They found significant odds of pancreatitis in ESRD patients (adjusted OR 1.7, p < 0.001) and any-stage CKD (adjusted OR 1.5, p < 0.001). It is notable however that the propensity score matching we employed in this study mitigates against selection bias better and likely provides a more precise estimate of the odds of post-ERCP pancreatitis in this patient population. When propensity score matching was applied to Kim et al. [14], the second study cited by the guidelines, they did not find a difference in the incidence of post-ERCP pancreatitis. This was notably a small study with only 39 patients in the dialysis group and the authors were not able to calculate an odds ratio. Park et al. [15] report the same frequency of PEP in patients on hemodialysis (5.4% to 10.3%) compared to the general population and a recent systematic review and meta-analysis comprehensively evaluating for predictors of PEP did not find that chronic kidney disease increases the risk of PEP [16]. Our data therefore suggests that the association between advanced chronic kidney disease and post-ERCP pancreatitis may have previously been overstated. This is clinically relevant, as extra potentially harmful precautions such as increased intravenous fluid administration which can lead to poor outcomes, may not be necessary in this patient population.

Post-procedural bleeding was noted in 2.9% of patients with aCKD and compared to the control group, aCKD patients had higher odds of post-ERCP bleeding. This finding was similar to other studies that evaluated post-ERCP AE in this high-risk population [7, 17] including a systematic review and pooled analysis by Park et al. [15] assessing morbidity and mortality associated with ERCP in hemodialysis (HD) patients which found a higher proportion of post-procedure bleeding in patients on hemodialysis (5.5%, 414/7544) compared with patients not on hemodialysis (1.5%, 6734/456,833, OR 3.84, 95% CI 4.26–25.5, p < 0.001). Multiple hypotheses have been suggested to explain the increased risk of bleeding in patients with aCKD including platelet dysfunction due to intrinsic platelet abnormalities and impaired platelet-vessel interactions [18, 19]. Other theories include having lower blood counts and the increased use of antiplatelet and anticoagulation agents in this population [20].

We found an increased odds of cholangitis in the advanced CKD cohort compared to the control cohort. To our knowledge, there is no other study that has previously assessed this particular ERCP-related adverse event in aCKD patients, so we do not have a comparator. However, we urge caution with the interpretation of this finding for a few reasons. First, in the general population, cholangitis has been estimated to occur in less than 3% of all patients undergoing an ERCP [5] but this is not the case in our data which shows a much higher than normal incidence of cholangitis even in our control cohort. One possible explanation is that available literature suggests patients with ESRD on dialysis have an increased overall risk of developing infections [21] so the development of post-procedure infectious symptoms from any cause might initially be attributed to cholangitis and coded as such due to temporal proximity to the ERCP. This then results in an over-estimation of cholangitis incidence. That being said, a study by Lee et al. [22] has demonstrated that patients with pre-existing renal dysfunction are at increased risk for multiorgan dysfunction in acute cholangitis, leading to increased morbidity and mortality. Hence, until this finding is negated by future studies, it is not unreasonable to pursue meticulous intraprocedural biliary drainage of contrast after all interventions are done and to consider consistent use of periprocedural antibiotics for now. With respect to our last primary outcome, we also found a similar odds of perforation between our aCKD cohort and the control cohort, which aligns with what the current literature suggests [15].

Our results for secondary outcomes suggest increased odds of ICU admissions, failure to extubate or new intubations within 48 h post ERCP, and all-cause mortality in our aCKD cohort compared to the control cohort. The proportion of 30-day all-cause mortality in the aCKD population of 6.9% is also higher than in previously published data [15]. Again, this is another finding we recommend interpreting with caution as the database does not allow for more granular analysis to determine the driving etiology for each of these poor outcomes and whether these poor outcomes are directly related to the ERCP procedure itself or the population of interest who tend to have significant comorbidities. This in turn means we are unable rule out confounders of this presumed association. However, if these findings are corroborated by future studies, then one possibility is volume overload in these patients may be a driving factor for the higher-than-expected odds of failed post-procedure extubations, new intubations and intensive care unit admissions. Periprocedural intravenous fluid administration is the norm while performing ERCPs and while the rate of administration is typically reduced in patients with advanced renal dysfunction, these patients may still receive a larger than anticipated fluid volume especially if they undergo prolonged procedures. Until these findings are categorically disproved, it may be prudent to coordinate non-emergent procedures with dialysis sessions in those who are already on dialysis.

Subgroup analysis revealed so significant differences in adverse events between the two cohorts except for ICU admissions 7 days post-procedure. This suggests that the progression from CKD4 to ESRD does not substantially alter the overall risk profile. While the rate of these complications does not differ, we do not know if the severity is higher in the ESRD group. Dialysis patients may be more vulnerable to hemodynamic instability, sepsis or metabolic derangements requiring intensive care. Hence, we would recommend coordinating non-emergent cases around hemodialysis sessions to manage volume/electrolytes and reduce the odds of ICU admissions.

The strengths of our study are a large, nationally representative sample size and the use of propensity score matching to mitigate against selection bias. This study has several inherent limitations. This is a retrospective cohort study. There may be residual unmeasured confounders which propensity score matching cannot mitigate against. Causality cannot be established for our positive results. The timing of holding and resumption of different platelet and anticoagulation agents was not captured in the data available to us, which could have influenced the odds of increased bleeding following ERCP. Like any database-based study, this research carries inherent concerns regarding possible diagnostic errors, incomplete documentation of certain variables, and inaccuracies in recorded diagnoses and ICD-10 codes. Additionally, we cannot assess whether hospital volume and individual operator experience would affect outcomes given the database is de-identified. Finally, complications might be underreported, especially if patients sought treatment outside the participating network, which is particularly relevant for post-procedure complications.

## Conclusion

In conclusion, aCKD is associated with higher odds of ERCP related adverse events with the notable exception of post-ERCP pancreatitis (PEP), which had similar odds to the control cohort, a finding that may change the previous widespread perception of increased PEP risk in this population. Further prospective research is needed to validate our findings.

## Supplementary Information

Below is the link to the electronic supplementary material.

Supplementary file1 (DOCX 17 kb)

## Abbreviations

ERCP: Endoscopic retrograde cholangiopancreatography
PEP: Post-ERCP pancreatitis
aCKD: Advanced chronic kidney disease
CKD4: Chronic kidney disease, stage 4
CKD5: Chronic kidney disease, stage 5
ESRD: End-stage renal disease
