# Supplementary material for: Advanced chronic kidney disease increases the odds of ERCP adverse events but not post-ERCP pancreatitis: a propensity-matched analysis of the US Collaborative Network
Source: Surg Endosc. 2025 Oct 22;40(1):404–12. doi: 10.1007/s00464-025-12323-x (PMC12823648; doi:10.1007/s00464-025-12323-x)
Supplement: Supplementary file 1 — Supplementary file1 (DOCX 17 kb) [file 464_2025_12323_MOESM1_ESM.docx]

**Supplementary Table 1: Data sources and diagnosis codes used for patient selection and outcomes**

| Terms | ICD-10, CPT, and RxNorm codes |
| --- | --- |
| Dependence on renal dialysis | Z99.2 |
| End stage renal disease | N18.6 |
| Chronic kidney disease, stage 5 | N18.5 |
| Chronic kidney disease, stage 4 (severe) | N18.4 |
| Chronic kidney disease, stage 3 (moderate) | N18.3 |
| Chronic kidney disease, stage 2 (mild) | N18.3 |
| Chronic kidney disease, stage 1 | N18.1 |
| Endoscopic retrograde cholangiopancreatography (ERCP); with sphincterotomy/papillotomy | 43262 |
| Endoscopic retrograde cholangiopancreatography (ERCP); with removal of calculi/debris from biliary/pancreatic duct(s) | 43264 |
| Endoscopic retrograde cholangiopancreatography (ERCP); with destruction of calculi, any method (e.g., mechanical, electrohydraulic, lithotripsy) | 43265 |
| Endoscopic retrograde cholangiopancreatography (ERCP); with placement of endoscopic stent into biliary or pancreatic duct, including pre- and post-dilation and guide wire passage, when performed, including sphincterotomy, when performed, each stent | 43274 |
| Endoscopic retrograde cholangiopancreatography (ERCP); with removal of foreign body(s) or stent(s) from biliary/pancreatic duct(s) | 43275 |
| Endoscopic retrograde cholangiopancreatography (ERCP); with removal and exchange of stent(s), biliary or pancreatic duct, including pre- and post-dilation and guide wire passage, when performed, including sphincterotomy, when performed, each stent exchanged | 43276 |
| Endoscopic retrograde cholangiopancreatography (ERCP); with trans-endoscopic balloon dilation of biliary/pancreatic duct(s) or of ampulla (sphincteroplasty), including sphincterotomy, when performed, each duct | 43277 |
| Acute pancreatitis | K85 |
| Melena | K92.1 |
| Hematemesis | K92.0 |
| Gastrointestinal hemorrhage, unspecified | K92.2 |
| Perforation of intestine (nontraumatic) | K63.1 |
| Accidental puncture and laceration of a digestive system organ or structure during a digestive system procedure | K91.71 |
| Critical Care Services | 1013729 |
| Cholangitis | K83.0 |
| Intubation, endotracheal, emergency procedure | 31500 |

*Abbreviations: RxNorm – A standardized terminology system for medications, ICD-10 – International Classification of Diseases, 10^th^ revision, CPT – Current procedural terminology*
